# Temporal Variation in Fish Mercury Concentrations within Lakes from the Western Aleutian Archipelago, Alaska

**DOI:** 10.1371/journal.pone.0102244

**Published:** 2014-07-16

**Authors:** Leah A. Kenney, Collin A. Eagles-Smith, Joshua T. Ackerman, Frank A. von Hippel

**Affiliations:** 1 Department of Biological Sciences, University of Alaska Anchorage, Anchorage, Alaska, United States of America; 2 U.S. Geological Survey, Forest and Rangeland Ecosystem Science Center, Corvallis, Oregon, United States of America; 3 U.S. Geological Survey, Western Ecological Research Center, Dixon, California, United States of America; University of Vigo, Spain

## Abstract

We assessed temporal variation in mercury (Hg) concentrations of threespine stickleback (*Gasterosteus aculeatus*) from Agattu Island, Aleutian Archipelago, Alaska. Total Hg concentrations in whole-bodied stickleback were measured at two-week intervals from two sites in each of two lakes from June 1 to August 10, 2011 during the time period when lakes were ice-free. Across all sites and sampling events, stickleback Hg concentrations ranged from 0.37–1.07 µg/g dry weight (dw), with a mean (± SE) of 0.55±0.01 µg/g dw. Mean fish Hg concentrations declined by 9% during the study period, from 0.57±0.01 µg/g dw in early June to 0.52±0.01 µg/g dw in mid-August. Mean fish Hg concentrations were 6% higher in Loon Lake (0.56±0.01 µg/g dw) than in Lake 696 (0.53±0.01 µg/g dw), and 4% higher in males (0.56±0.01 µg/g dw) than in females (0.54±0.01 µg/g dw). Loon Lake was distinguished from Lake 696 by the presence of piscivorous waterbirds during the breeding season. Mercury concentrations in stickleback from Agattu Island were higher than would be expected for an area without known point sources of Hg pollution, and high enough to be of concern to the health of piscivorous wildlife.

## Introduction

Mercury (Hg) is a toxic metal that is globally distributed from both natural and anthropogenic sources [1]. Inorganic Hg is converted to the highly toxic form methlymercury (MeHg) by microbial methylation and assimilated into aquatic food webs through the processes of bioaccumulation and biomagnification [2]. Due to the residence time of elemental Hg in the atmosphere (several months to a year), it can be transported considerable distances, which results in enhanced Hg deposition in locations that are far removed from Hg point sources [3], [4].

**Figure 1:**
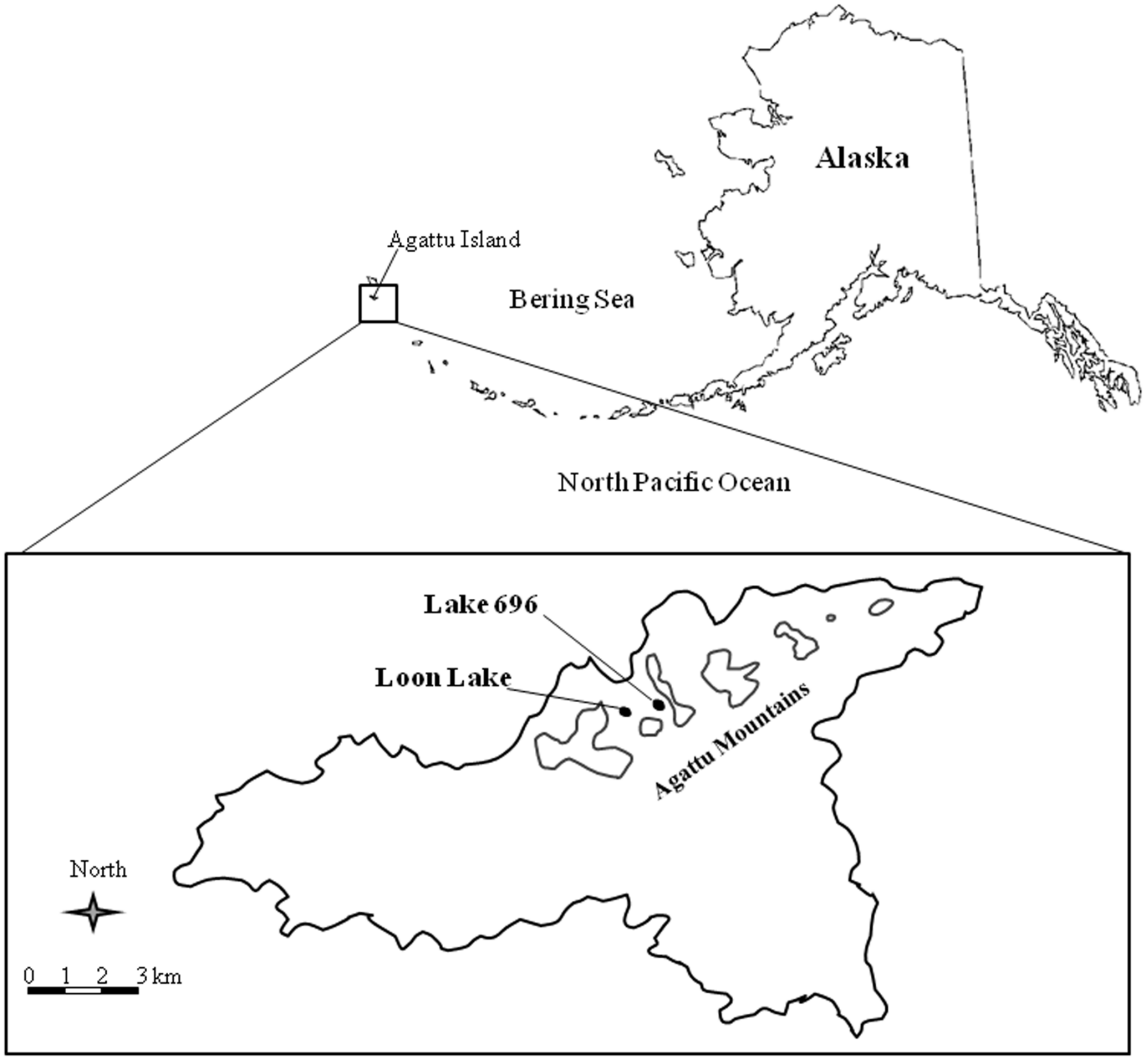
Location of study at Agattu Island in the Aleutian Archipelago, Alaska. Outline of Agattu Mountains is indicated by 300

The Aleutian Archipelago (hereafter, Aleutians) is an isolated arc of >300 volcanic islands stretching 1,600 km and separates the North Pacific Ocean from the Bering Sea (Fig. 1). Despite the remoteness of the Aleutians, elevated concentrations of contaminants, including Hg, have been documented in marine organisms throughout the archipelago. Military activities at some islands have been implicated as sources of Hg contamination [5]–[7], but elevated Hg concentrations in biota collected near islands with no military history have also been documented [8]–[13].

The Aleutians are a critical region for avian conservation, supporting more than 10 million seabirds, as well as sea ducks and other birds [14]. Many of these birds rely on freshwater ecosystems within the islands for roosting, foraging, and breeding sites. Elevated concentrations of Hg in forage fish can pose health consequences for fish-eating wildlife through reproductive impairment and neurotoxic effects [15], [16]. Little is known about Hg pollution in freshwater habitats within the Aleutians, but they may serve as sites for Hg exposure for piscivorous wildlife.

Establishing a contemporary record of Hg levels in these remote islands could be important for documenting long-term trends. Although the global pool of atmospheric Hg is predicted to increase in association with on-going industrial development and subsequent emissions throughout the world [17], the U.S. Environmental Protection Agency (EPA) will soon implement the Mercury and Air Toxics Standards rule which will require up to a 90% reduction in Hg emissions from all coal and oil-powered electrical generators in the U.S. [18]. Monitoring in the North Pacific, a region likely influenced by global industrial activity, is key for understanding aquatic ecosystem responses to changes in global Hg emissions and deposition [19]. Furthermore, non-migratory freshwater species provide an ideal model for spatial and temporal monitoring of long-term trends in Hg exposure within this region.

Our objectives were two-fold. First, we quantified Hg concentrations in a freshwater forage fish at a remote island in the Aleutian Archipelago that has no history of local anthropogenic Hg contamination. Second, we examined whether Hg concentrations in fish varied temporally, by assessing their concentrations at two sites each within two lakes on the isolated island.

We used threespine stickleback (*Gasterosteus aculeatus*; hereafter stickleback) as a lower trophic level bioindicator of Hg exposure in freshwater lakes. Stickleback are a generalist consumer found throughout much of the northern hemisphere and are the most widespread and abundant freshwater fish in the Aleutians [20]. Stickleback are an important prey resource for waterbirds, including red-throated loon (*Gavia stellata*), a species of conservation concern in this region [21]. Furthermore, stickleback have been used successfully as a biomonitoring species for Hg in both the Aleutians [22] and elsewhere [23], [24].

## Materials and Methods

Ethics Statement: All collections during this study were done in accordance with and approved by the University of Alaska Anchorage Institutional Animal Care and Use Committee (protocol 159870-11) and were authorized by the Alaska Department of Fish and Game (permit SF2011-029).

### Study area

Agattu Island (52.43° N, 173.60° E) is located at the western end of the Aleutian Archipelago (Fig. 1) within the Alaska Maritime National Wildlife Refuge. The island covers an area of 22,474 ha composed primarily of low elevation (<200 m) broad rolling plateaus and abundant wetlands. We studied two lakes on Agattu Island that have similar morphometry, ecology, and climate, but that differed in their use by waterbirds. Loon Lake was commonly used by glaucous-winged gull (*Larus glaucescens*) for roosting, and by red-throated loon and common loon (*G. immer*) for roosting, foraging, and breeding throughout the summer months (June-August), whereas no waterbirds were observed at Lake 696 during the same time period.

The two lakes have similar estimated surface areas (Loon Lake = 1.5 ha, Lake 696 = 1.2 ha), which are typical for lakes on Agattu Island. The northern portions of both lakes are comprised of bedrock substrate that descends steeply into deeper waters (2–5 m), whereas the southern sides are shallow (<1 m) with substrates composed of finer sediments. Each lake has an ephemeral outlet stream along the north shore, from which sufficient water had evaporated by the first trapping effort to isolate the outlet stream from the lake itself. Thus, there was no immigration or emigration of stickleback during the study period.

As part of the expanded military presence in the Aleutians during World War II, a military radar installation operated from 1943–1946 at the southeast corner of Agattu Island. The radar station occupied a relatively small (<1000 ha) and localized area and none of our sampling took place within this area. Thus, it is unlikely that the radar station at Agattu Island influenced Hg values of the present study.

### Field collections

Stickleback were collected every two weeks from June 1 to August 10, 2011 for a total of six trapping efforts at each lake. Our sampling period corresponded with the ice-free season, which is generally from May-October in the western Aleutians (1949–2005; Western Regional Climate Center) and during the reproductive season for both stickleback and piscivorous waterbirds (June-August). Stickleback were trapped using unbaited 0.6 cm mesh minnow traps set at 0.5–2 m depth for ≤24 hours. Six traps were placed along the north side of each lake near the mouth of the outlet stream, and another six traps were placed along the south side of each lake. A random subset of 15 stickleback from the north and south sides of each lake at each trapping effort were collected for a total of 360 fish. Stickleback were euthanized using pH neutral MS-222 anesthetic. Due to the remote location of the study site and lack of refrigeration, fish were rinsed in lake water and then placed in 95% undenatured ethanol for tissue fixation and preservation. After 48 hours, the ethanol was replaced to further prevent postmortem decay of fish tissues.

### Sample preparation

Upon return to the laboratory, standard length (anterior tip of premaxilla to posterior border of hyperal plate) of each fish was measured with digital calipers (±0.01 mm). Stomach contents were removed with stainless steel forceps and discarded, and the stomach was placed back into the cavity of the fish. Following removal of stomach contents, whole body mass was obtained ±0.001 g. Sex was determined based on inspection of gonads.

Fish were oven dried at 50°C for approximately 48 hours. Dried samples were homogenized by cryogenic (liquid nitrogen immersion) grinding using a cryogenic impact mill (6770 Freezer Mill; Spex, Metuchen, NJ, USA) and placed in sterile 25 mL vials. Homogenized samples were placed in the drying oven at 50°C for approximately 48 hours to remove any residual moisture obtained during cryogenic grinding.

### Mercury analysis

Previous research has demonstrated that >90% of Hg in fish tissues is in the MeHg form [25], suggesting that total Hg (THg) is an adequate surrogate for MeHg. Following U.S. EPA method 7473 [26], we determined THg concentrations for each fish on a Milestone Direct Mercury Analyzer (DMA-80, Milestone Inc). Analytical equipment was calibrated using certified standard solutions prior to analysis, and accuracy and precision were evaluated within each analytical batch through the inclusion of certified reference materials (either dogfish muscle tissue [DORM-3] or dogfish liver [DOLT-4] from the National Research Council of Canada), calibration verifications (liquid standards), duplicates, and blanks. Recoveries averaged 103.26%±1.43% (*n* = 26) and 94.62%±1.38% (*n* = 38) for certified reference materials and calibration checks, respectively. Absolute relative percent difference for all duplicates averaged 1.14%±0.21% (*n* = 24).

Hg data are presented on a dry weight (dw) basis. The mean (± SE) percent moisture for un-preserved Aleutian stickleback is 77.1%±0.024% [20]; this percent moisture can be used to facilitate comparisons with other studies in which only wet weight (ww) Hg values were reported.

### Statistical methods

All analyses were conducted using JMP v.10.0 statistical software (SAS Institute). Mercury data were natural-log transformed to normalize residuals and to reduce heteroscedasticity. We evaluated the effects of date (day of year), lake, sex, and capture site within each lake (north or south), and fish standard length using a linear mixed-effects model. Potential non-linear changes in fish Hg concentrations were evaluated by including day of year as both a linear and quadratic variable (date^2^). In order to structure the model within a repeated measures framework, we also included a random effect variable constructed from the variables: lake, site, and date. We used the likelihood ratio test to assess the relative fit of the linear vs. quadratic date models, as well as a model with and without a lake × date interaction. We present results as back-transformed least squares means from the final global model with standard errors unless otherwise specified.

## Results

Fish sizes were similar between lakes, with standard lengths ranging between 39.2–63.2 mm (mean  = 47.5 mm, *n* = 180) and 38.9–60.2 mm (mean  = 46.0 mm, *n* = 180) at Loon Lake and Lake 696, respectively. On the first sampling date only, there were unusually large fish (range  = 54.0–63.2 mm, *n* = 12) that were likely adult survivors of the previous season and hence a different age cohort. In order to reduce the likelihood of older fish influencing our temporal results, these individuals were removed from analyses. Across all sites and sampling events, stickleback THg concentrations ranged from 0.37–1.07, with a mean (± SE) of 0.55±0.01 µg/g dw.

**Figure 2:**
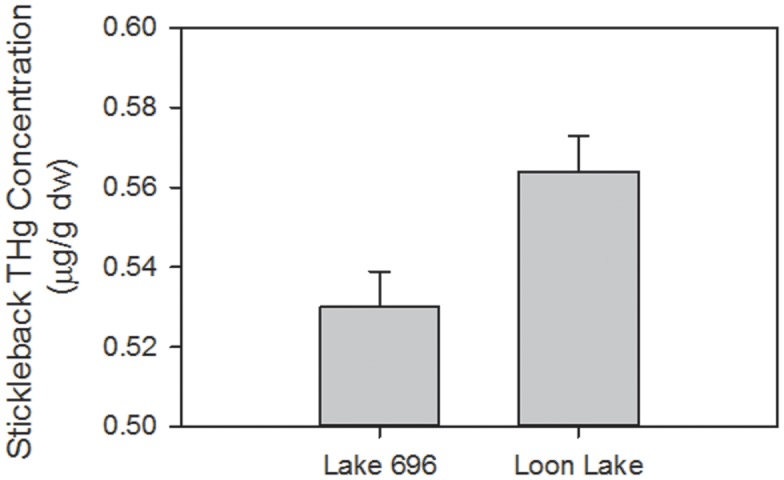
Total mercury (THg) concentrations in threespine stickleback collected from two lakes on Agattu Island in the Aleutian Archipelago, Alaska, 2011. Concentrations are reported as back-transformed least square means from the global mixed-effects model which accounts for other variables. Error bars represent ± SE.

**Figure 3:**
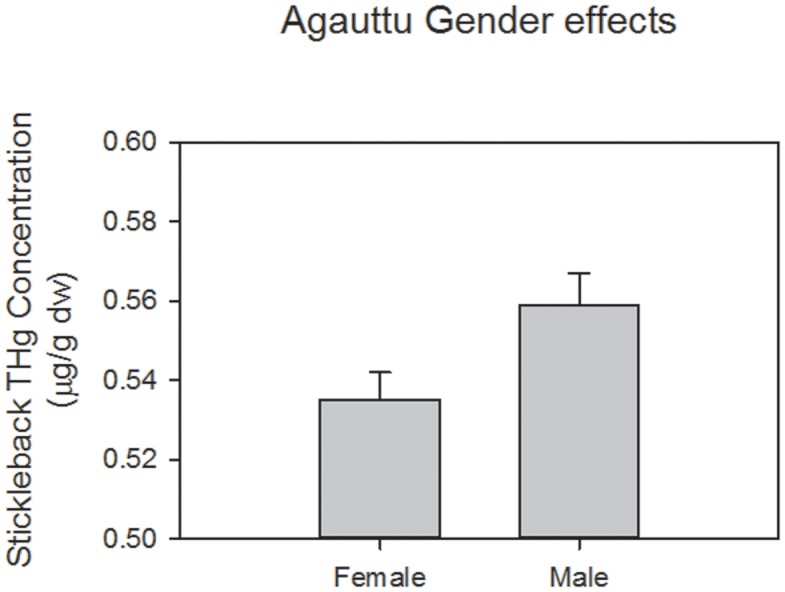
Total mercury (THg) concentrations in female and male threespine stickleback collected at two lakes at Agattu Island, Aleutian Archipelago, Alaska, 2011. Concentrations are reported as back-transformed least square means from the global mixed-effects model which accounts for other variables. Error bars represent ± SE.

**Figure 4:**
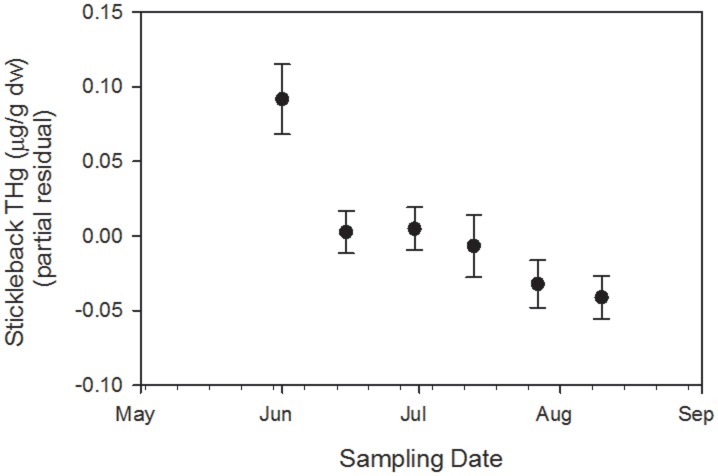
Partial residuals of total mercury (THg) concentrations (± SE) in threespine stickleback collected bi-monthly from two lakes at Agattu Island, Aleutian Archipelago, Alaska, 2011. The partial residuals account for other variables in the global mixed-effects model.

The likelihood ratio test indicated that the mixed-effects repeated measures model with linear and quadratic date components was not a better fit than the model with just the linear date form (*n* = 348, χ^2^ = 1.02, *p* = 0.31). Similarly, the model with the site × date interaction did not fit better than the model without this interaction (*n* = 348, χ^2^ = 0.59, *p* = 0.44). Therefore, our final model contained the fixed effects of lake, sampling site within lake, standard length, day-of-year, sex, and the random effects variable described above. Using this final model, we found that THg concentrations varied between lakes (F_1,344_ = 6.93, *p*<0.02; Fig. 2), sexes (F_1,344_ = 7.37, *p*<0.01; Fig. 3), and date (F_1,344_ = 8.99, *p*<0.01; Fig. 4), but did not vary between sites within lakes (F_1,344_ = 2.24, *p* = 0.15) or by fish standard length (F_1,344_ = 2.52, *p* = 0.11). There was a 9% decrease in fish THg concentrations across the study period, from a mean of 0.57±0.01 µg/g dw in early June to 0.52±0.01 µg/g dw in mid-August (Fig. 4). Fish THg concentrations were 6% higher in Loon Lake (0.56±0.01 µg/g dw) than in Lake 696 (0.53±0.01 µg/g dw), and were 4% higher in males (0.56±0.01 µg/g dw) than in females (0.54±0.01 µg/g).

## Discussion

Mercury concentrations in stickleback residing in freshwater lakes on Agattu Island were relatively high considering the remote location of the island and its lack of any known direct anthropogenic Hg contamination. We found that Hg concentrations in stickleback from Agattu Island are substantially higher (0.55 µg/g dw) than other stickleback populations from mainland Alaska that also lack point sources of Hg pollution (southwest Alaska 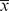 = 0.11 µg/g dw, *n* = 30, [27]; southcentral Alaska 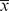 = 0.17 µg/g dw, *n* = 159 [24]). Furthermore, Hg concentrations in stickleback from Agattu Island were comparable to or higher than mean stickleback Hg concentrations from regions in Poland (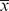 = 0.18 µg/g ww [28]; 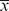 = 0.01–0.49 µg/g ww [29]) and California (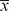 = 0.40–0.52 µg/g dw [23]) that are near known sources of Hg contamination.

We observed a significant decline in fish Hg concentrations over the course of the summer, although the magnitude of change over time was modest (9%). We also found small but statistically significant differences in fish Hg concentrations between lakes (Hg concentrations were 6% higher in Loon Lake than Lake 696) and between sexes (Hg concentrations were 4% higher in males than females).

Although Hg concentrations in stickleback from Agattu Island were higher than would be expected for an area without a known point source of Hg pollution, other studies have documented comparatively high concentrations of contaminants in marine organisms from the western Aleutians [8]–[10], [13], [30]. Recent work at Adak Island, located in the central Aleutians, also revealed similarly high mean Hg concentrations in resident freshwater stickleback populations (range  = 0.31–0.54 µg/g dw) [22]. The Aleutian Low, a semi-permanent low-pressure system characterized by many strong cyclones, dominates weather patterns of the North Pacific and Bering Sea. These cyclones pull storms, and presumably atmospheric contaminants, from lower latitudes and Asia eastward along the archipelago where contaminants can condense and precipitate out of the atmosphere [3]. As a consequence, the Aleutians may receive higher rates of atmospheric deposition of inorganic Hg than elsewhere in Alaska.

Although atmospheric transport of Hg derived from fossil fuel combustion is likely the primary pathway of Hg to arctic regions [4], other possible non-point sources of Hg contamination in the Aleutians include volcanic activities and biotransport. Volcanic events release inorganic Hg that can enter aquatic ecosystems and be methylated into the more toxic and bioavailable MeHg. Although islands in the far western Aleutians lack active volcanoes, the Kamchatka Peninsula in Russia (∼350 km west of Agattu Island) is a volcanically active area and thus the contribution of volcanic Hg from Kamchatka in addition to other island groups within the Aleutians could be an additional source of Hg.

Biological transport of Hg to freshwater systems has been documented in seabirds [22], [31], [32]. Although the difference was relatively small, we found that fish Hg concentrations were 6% higher in Loon Lake (which was consistently used by waterbirds) than in Lake 696 (where waterbirds were never observed). Given their widespread use of lakes at Agattu Island and across the Aleutian Archipelago [20], seabirds may play a role in the biotransport of Hg between marine and freshwater habitats in this region. Further study is needed to quantify the relative importance of this mechanism.

A growing body of literature has focused on identifying temporal variation in fish Hg concentrations in order to appropriately design monitoring programs [23], [33], [34]. We also found temporal variability in Hg concentrations of stickleback sampled at Agattu Island. Similar to our findings, fish Hg concentrations were generally higher during late spring or early summer and lowest during late summer and fall [23], [33]–[35]. There are several potential mechanisms that could explain the higher Hg values documented in stickleback collected earlier in the summer, such as enhanced MeHg production associated with rising temperatures or Hg delivery via snowmelt, but examining mechanistic drivers was beyond the scope of this study.

Although the magnitude of temporal variation in fish Hg concentrations that we documented at Agattu Island was modest, sampling at just one time period during our study (e.g., in June or August; Fig.4) would have inaccurately characterized fish Hg concentrations throughout the summer and could have underestimated or overestimated the exposure risk to piscivorous wildlife [23]. At a minimum, these temporal studies indicate that Hg monitoring programs need to carefully consider the timing of sampling in order to be robust.

The breeding season is among the most vulnerable periods for Hg exposure for piscivorous waterbirds [23], [36]. Mercury concentrations in stickleback from both lakes (adjusted to wet weight [ww] values: Loon Lake: 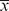 = 0.13 µg/g ww; Lake 696: 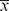 = 0.12 µg/g ww) fell within the range of dietary concentrations associated with deleterious endpoints. Specifically, 93% of the prey fish sampled at Agattu Island exceeded a proposed benchmark for behavioral impairment (0.10 µg/g ww) in common loons, although only 1% exceeded the proposed benchmark for reproductive impairment (0.18 µg/g ww [37]). Similarly, 100% of fish sampled exceeded thresholds associated with impaired biochemical (0.06 µg/g ww) and reproductive (0.04 µg/g ww) endpoints in predatory fishes, but none of the stickleback exceeded thresholds associated with altered behavior (0.50 µg/g ww) or growth (1.44 µg/g ww) in fishes [38].

Mercury concentrations in stickleback from all time periods were relatively high. Thus, stickleback could be an important dietary source of Hg for waterbirds that forage in these lakes throughout the summer. Our findings highlight the potential for Hg exposure to the millions of waterbirds that nest and forage within freshwater lakes in the Aleutians. Future monitoring at a broader scale (more lakes and more islands within the archipelago) is warranted to further elucidate the impacts of Hg exposure to piscivorous bird populations in this region.

When using fish as biosentinels in Hg monitoring programs, it is imperative to evaluate the effects of co-variables that may confound the interpretation of results [39]. In addition to date and lake effects, we also found that males had approximately 4% higher Hg concentrations than females (Fig. 3). There are several potential mechanisms that could contribute to this small difference in Hg concentrations between sexes. During the breeding season, females consume high caloric foods, including fish eggs, and generally undergo an increase in body mass [40]. Consequently, females may reduce their Hg tissue concentration through growth dilution [41], while simultaneously offloading Hg into their eggs. In contrast, males engage in energetically demanding activities such as defending their territory and tending the nest which likely contribute to a loss in body mass, and consequently an increase in tissue concentration of Hg [41]. The specific factors driving variation in Hg concentrations between males and females at Agattu Island remain unclear, but could be a combination of differences in diet, maternal transfer to eggs, and the process of growth dilution.

## Conclusions

The elevated concentrations of Hg in stickleback populations from Agattu Island within the Aleutian Archipelago illustrate the need for further study of Hg pollution in this remote region. The present study highlights parameters useful for management and biomonitoring decisions, especially temporal variations in fish Hg concentrations. Specifically, sampling date should be considered when monitoring Hg concentrations in fish and the timing of sampling should focus on the breeding season when piscivorous waterbirds or other predators are at greatest risk of Hg exposure [23]. This is especially true in the Aleutians where over 10 million waterbirds breed annually, and Hg concentrations in stickleback, the most common freshwater fish, were found to exceed known impairment guidelines for birds.

## Acknowledgments

Invaluable logistical support was provided by the Alaska Maritime National Wildlife Refuge, especially from Jeff Williams, Lisa Spitler, Heather Renner and the crew of the R/V Tiglax. We thank Robb Kaler for assistance with study design and field collections. We thank Kyle Shedd, Robin Keister, Mark Herzog, Branden Johnson, and Brandon Kowalski for laboratory assistance. We thank Robb Kaler, Mark Ricca, Emilie Saulnier-Talbot, and an anonymous reviewer for constructive feedback and comments that improved this manuscript. All collections during this study were done in accordance with the University of Alaska Anchorage Institutional Animal Care and Use Committee (protocol 159870-11) and were authorized by the Alaska Department of Fish and Game (permit SF2011-029). Any use of trade, product, or firm names is for descriptive purposes only and does not imply endorsement by the U.S. Government.
